# Acknowledgement to the reviewers for 2021

**DOI:** 10.1590/2175-8239-JBN-2022-P001

**Published:** 2022-02-17

The Editor-in-Chief and Associated Editors of the **Brazilian Journal of Nephrology** would like to thank all **178 individuals** who served as reviewers in 2021 and put their time and effort into analyzing the submitted manuscripts to ensure that only the best submitted papers are published in the journal.

**Reviewers**

Abel Botelho Quaresma

Abhijeet Saha

Adriano Ammirati

Alba Otoni

Alejandro Marín

Alessandra Becker-Finco

Alexandre Araujo

Alexandre Bignelli

Alexandre Silvestre Cabral

Aluizio B. Carvalho

Alvaro Silva Filho

Amanda Rabello Crisma

Ana Cristina Simões e Silva

Ana Elizabeth Figueiredo

Ana Moura

Ana Ravazzani

André Balbi

Andre Pereira

Andrea Carla Bauer

Andrea Emília Stinghen

Andreia Watanabe

Angélica Adamoli

Arsh K. Jain

Barbara Vogt

Beatriz Reis

Bengt Lindholm

Bruno Adler Maccagnan Pinheiro Besen

Bruno Cabrita

Bruno Eduardo Balbo

Caio Fernandes

Caio Pellizzari

Camila Eleuterio

Carlos Gomes

Carlucci Ventura

Carmen Tzanno

Cassiana Goes

Chris Karohl

Cibele Rodrigues

Clotilde Garcia

Cristiane Dias

Cristianne Alexandre

Daiana Bündchen

Daniela Ponce

Danielle Ezequiel

David Machado

Dayana Bitencourt

Dirceu Silva

Domingos Chula

Domingos Otávio D'Avila

Ebru Asicioglu

Edison Souza

Edmundo Lopes

Edneia Cavalieri

Eduardo Rocha

Elieser Watanabe

Elis Pedrollo

Elisa Azevedo Souza

Elizabeth Daher

Elizete Keitel

Emmanuel Burdmann

Erika Rangel

Eyup Altinoz

Fabiana Bonato

Fabiana Nerbass

Fernanda Gorayeb-Polacchini

Fernando Almeida

Fernando Luiz Affonso Fonseca

Flanklin Barcellos

Flávio Farias Filho

Francisco José Veronese

Geraldo Silva Junior

Gina Gordon

Gisele Meinerz

Hélady Sanders-Pinheiro

Henrique Takase

Ita Heilberg

João Saldanha de Castro Filho

Jocemir Ronaldo Lugon

José Alberto Pedroso

Jose Carolino Divino Filho

Jose Leonardo Faustini Pereira

José Moura-Neto

José Suassuna

Juliana Mansur

Juliana Oliveira

Juliano Ferreira Arcuri

Khaled Boubes

Laila Andrade

Ligia Pierrotti

Lilian Palma

Luciane Deboni

Luciano Selistre

Luciano Wolffenbuttel

Lúcio Roberto Requião-Moura

Luis Andrade

Luis Fernando Onuchic

Luis Gustavo Modelli de Andrade

Luis Martin

Luiz Nakamura

Luiza Jane Eyre Souza Vieira

Manuel Castro

Marcela Sorelli

Marcele Alves

Marcelo Bacci

Marcelo Nascimento

Marcelo Pinheiro

Marcelo Tavares

Marcia Franco

Marcos Sousa

Marcus Bastos

Maria Almerinda Ribeiro-Alves

Maria Cristina Andrade

Maria de Fatima Vattimo

Maria Goretti Penido

Marilda Mazzali

Mario Bonomini

Maristela Bohlke

Marlene Reis

Mateus Dias Antunes

Mauricio Carvalho

Maurício Younes-Ibrahim

Maurilo Leite Júnior

Maurizio Gallieni

Maycon Reboredo

Melani Custodio

Murilo Guedes

Nara Costa

Natalia Knoll

Natália Maria Fernandes

Nicola Wearne

Niels Camara

Nilzete Bresolin

Paula Chiarello

Paula Presti

Paulo Batista

Paulo Gregório

Paulo Nogueira

Paulo Suassuna

Peter Blake

Precil Neves

Rachel Armani

Rafael Weissheimer

Ramón Paniagua

Rebecca J. Johnson

Rejane Bernardes

Renato Foresto

Roberto Manfro

Rodrigo de Oliveira

Rodrigo Hagemannn

Rogerio Paula

Rolando Claure-Del Granado

Rosana Shuhama

Rosilene Elias

Rubens Lodi

Rui Barros

Samile Sallaberry

Samirah Gomes

Samuel N. Uwaezuoke

Sérgio Bucharles

Sergio Yamada

Silvia Ribeiro

Silvia Rodriguez

Simone Appenzeler

Sophie Liabeuf

Stanley Araujo

Tainá Sandes-Freitas

Thais Gascon

Thyago Moraes

Valter Garcia

Vanessa Silva

Vera Hermina Koch

Verônica Antunes

Vinicius Delfino

Viviane Calice-Silva

Viviane Souza

Wesley Souza

